# *EGFR* Status Assessment for Better Care of Early Stage Non-Small Cell Lung Carcinoma: What Is Changing in the Daily Practice of Pathologists?

**DOI:** 10.3390/cells10082157

**Published:** 2021-08-21

**Authors:** Paul Hofman

**Affiliations:** 1 Laboratory of Clinical and Experimental Pathology, CHU Nice, FHU OncoAge, Pasteur Hospital, Université Côte d’Azur, 06108 Nice, France; hofman.p@chu-nice.fr; Tel.: +33-492-038-855; Fax: +33-492-8850; 2 CHU Nice, FHU OncoAge, Hospital-Integrated Biobank BB-0033-00025, Université Côte d’Azur, 06000 Nice, France

**Keywords:** non-small cell lung carcinoma, early stage, targeted therapies, *EGFR*, molecular tests

## Abstract

The recent emergence of novel neoadjuvant and/or adjuvant therapies for early stage (I-IIIA) non-small cell lung carcinoma (NSCLC), mainly tyrosine kinase inhibitors (TKIs) targeting *EGFR* mutations and immunotherapy or chemo-immunotherapy, has suddenly required the evaluation of biomarkers predictive of the efficacy of different treatments in these patients. Currently, the choice of one or another of these treatments mainly depends on the results of immunohistochemistry for PD-L1 and of the status of *EGFR* and *ALK*. This new development has led to the setup of different analyses for clinical and molecular pathology laboratories, which have had to rapidly integrate a number of new challenges into daily practice and to establish new organization for decision making. This review outlines the impact of the management of biological samples in laboratories and discusses perspectives for pathologists within the framework of *EGFR* TKIs in early stage NSCLC.

## 1. Introduction

Developments in the treatment of patients with advanced stage non-small cell lung carcinoma (NSCLC) have improved not only the overall quality of life but also the life expectancy of these patients [1,2]. For a long time, treatment of early stage NSCLC included only surgical resection, sometimes associated with radio-chemotherapy or neoadjuvant or adjuvant chemotherapy [3,4,5]. In fact, these associated treatments provided a moderate benefit in terms of progression-free survival and overall survival [3,4]. The recent results of adjuvant therapy targeting activating mutations in *EGFR* (exon 19 deletion and L858R mutation) and of preoperative immunotherapies of early stage (I-IIIA) NSCLC open up great perspectives for the prevention of relapse and/or post-operative tumor progression [6,7,8,9,10,11,12,13,14]. In this context, laboratories of pathology have been required to meet new challenges in developing a number of analyses to assure optimal care of patients in a routine clinical practice. Therefore, it is mandatory to look for different biomarkers, including PD-L1 assessment by immunohistochemistry (IHC), and *EGFR* status evaluation by molecular testing [15,16]. Currently, PD-L1 IHC in tissue samples is the only biomarker used routinely as a companion diagnostic test for the assessment of predictive immunotherapy and immunochemotherapy efficacy in thoracic oncology [15,16,17]. Thus, PD-L1 IHC was used as a predictive biomarker in clinical trial development for early stage NSCLC treated with immune checkpoint inhibitors (ICIs) [11,12,13]. After briefly presenting the main current clinical trials concerning the anti-*EGFR* targeted therapies in early stage NSCLC, this review will deal with the different steps for the setup and management of predictive tests that must be performed in laboratories for the care of these patients. Then, it will address the associated issues and constraints concerning this new opportunity.

## 2. Short Overview of Studies concerning the Use of *EGFR* TKIs in Early Stage NSCLC in Neoadjuvant and Adjuvant Setting

Different clinical trials have been performed with the aim to evaluate the efficacy of TKIs targeting activating mutations in *EGFR* in the neoadjuvant and also in the adjuvant setting [18,19,20]. Briefly, these trials have used or use inhibitors of the first, second, and third TKI generation [19,21]. Some of these trials have given relatively disappointing results, in particular for the first and second generation of TKIs, which have not provided a significant benefit to patients in terms of quality of life and absence of recurrence and overall survival [22]. However, recent studies, in particular the ADAURA study which used osimertinib, a third generation TKI, as an adjuvant for non-epidermoid NSCLC (stage IB-IIIA completely surgically resected) with an exon 19 deletion or an L858R mutation in *EGFR*, were positive compared to a placebo for progression-free survival [14]. Studies using other third generation TKIs (such as afatinib (NCT01746251 trial) or almonertib (NCT0468741 trial)) as an adjuvant after complete surgical resection are also ongoing. Clinical trials with other third generation TKIs targeting *EGFR* mutations in a neoadjuvant setting (such as the NEOADAURA study (comparison of treatment with osimertinib versus osimertinib and chemotherapy versus placebo and chemotherapy) or the afatinib trial (NCT04470076 trial)) have also been developed for early operable stages of NSCLC [23].

In this context, the positive results of the ADAURA study have recently led the Food and Drug Administration (FDA) and, subsequently, the European Medicines Agency (EMA) to approve the use of osimertinib for early stage NSCLC with the activating mutations defined above [24,25]. Thus, the systematic search for *EGFR* mutations in early stage non-squamous NSCLC has become obligatory in any pathology laboratory. Therefore, it is crucial to highlight that the current approval of osimertinib is restricted to the adjuvant therapy after complete tumor resection in adult patients with stage IB-IIIA non-squamous cell lung carcinoma harboring *EGFR* exon 19 deletions or exon 21 (L858R) substitution mutations [24,25]. Until now, these stages of I-IIIA lung cancer patients having other subtypes of genomic alterations, such as *EGFR* exon 18, non L858R exon 21 or T790M, and non T790M exon 20 mutations, could not be treated with osimertinib [24,25]. Moreover, it is noteworthy that the possibility of proposing, at an early time, targeted therapies oriented not only to these latter *EGFR* mutations but also to other genomic alterations in these early stages is presently being investigated, and it may also be soon that some of them are administrated in daily practice in the near future [26].

## 3. Which Biological Sample for Evaluation of the *EGFR* Status of Early Stage NSCLC?

**Figure 1:**
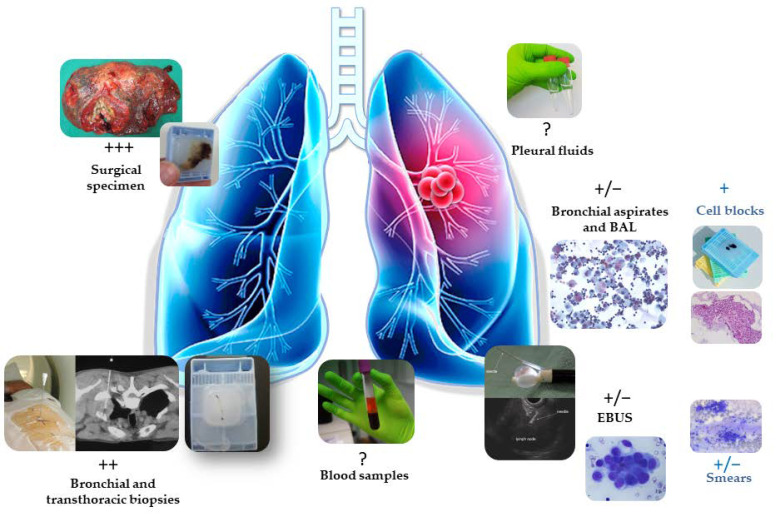
Different sources of biological specimen available for *EGFR* status assessment in early stage NSCLC. BAL, bronchoalveolar lavage; EBUS, endobronchial ultrasound biopsy. According to the samples, the assessment of genomic alteration can be globally more or less easy since the quantity of extracted nucleic acids is generally variable in these samples (+++: very high quantity; ++: high quantity; +: moderate quantity; +/−: low quantity; ?: uncertain quantity).

The *EGFR* status of tumors of patients with advanced stage NSCLC can be evaluated with several biological sources: (i) tissue biopsies (of bronchial or transthoracic origin); (ii) cytological samples (from transbronchial or transthoracic fine needle aspiration; fluid effusions, in particular pleural or cerebrospinal; and bronchoalveolar lavage); (iii) blood samples, mostly from circulating free DNA and sometimes from circulating tumor cells (CTCs); and, more rarely, (iv) surgical specimens [27] (Figure 1). All of these samples can also be analyzed in early stage NSCLC for genomic alteration assessment, though, at the moment, the majority probably consist of surgical specimens and, less frequently, biopsies. Thus, a number of questions can be asked. Notably, for adjuvant TKI, is it preferable to evaluate the preoperative status of *EGFR* on tissue biopsies, or is it better to look for the *EGFR* mutations on surgical specimens? In fact, preoperative assessment is certainly the ideal situation, knowing that it is necessary to obtain the *EGFR* and *ALK* status before envisaging neoadjuvant immunotherapy or neoadjuvant immunotherapy and chemotherapy [28]. Thus, the preoperative choice of therapy (with neoadjuvant and/or adjuvant administration of targeted molecules) must be made before surgery [28]. However, a number of limits may exist [29,30,31]. The size of the biopsy samples is more and more often small, in part due to the optimization of endoscopic techniques with the use of supple small caliber endoscopes that give access to distal tumors [32]. In this setting, molecular biological tests can give false negative results. This may be due to the extraction of an insufficient number of nucleic acids for sequencing analyses (notably due to a low percentage of tumor cells, below 20% among most recommendations, or to an extensive area of necrosis) and/or to a poor quality of these extracted nucleic acids (due to hyper- or hypo-fixation of the tissue or, very exceptionally, to a long period of cold ischemia time with a delay in fixation). Although controversial, false negative results for *EGFR* mutations can also result from tumor heterogeneity [33,34,35,36]. When this is the case and in the absence of an identifiable mutation, it is certainly necessary to systematically evaluate the *EGFR* status of the resected specimen. Though rare, mutations in *EGFR* can also be investigated with preoperative cytological samples, but negative results require secondary analysis of the mutational status of the surgical specimen [37,38]. The practice of liquid biopsies (LB) has strongly modified the care of advanced stage NSCLC patients, not only during progression but also, recently, at diagnosis [39,40,41,42,43]. Thus, LB can correspond to different fluids including blood samples but also pleural, pericardial, and ascites effusions or cerebrospinal fluid [44,45,46]. In particular, these different LBs can be used in advanced stage NSCLC patients to detect genomic alterations that are accessible to targeted therapies [39,40,41,42,43,44,45,46]. However, the sensitivity of blood samples for *EGFR* evaluation is globally low for solid tumors at an early stage of NSCLC, given the low number of free nucleic acids circulating in the blood and/or of the low number of CTC detections associated with an *EGFR* mutation [47,48]. Thus, preoperative evaluation of the *EGFR* status with blood samples is associated with a high percentage of negative results. Testing *EGFR* status from other LBs (notably from pleural effusions) is not useful since, by definition, pleural involvement is absent in early stages of NSCLC. When a very low amount of circulating tumor DNA is present in the plasma sample, certain technical approaches, but also better control of pre-analytical blood handling processes, could optimize detection of genomic alterations [49,50,51]. LB performed at different post-operative times can quantify the circulating tumor DNA and/or identify the presence of mutations in *EGFR*, which can point to recurrence or tumor progression [52]. Additionally, apart from CTCs and circulating tumor DNA, blood samples from lung cancer patients can contain other circulating components of strong interest, such as extracellular vesicles (EVs) [53,54,55,56]. Therefore, all cells, including tumor cells, can release EVs in the blood stream, which are broadly divided into ectosomes and exosomes. More and more studies have focused on exosomes’ assessment in LB because of their potential not only in lung cancer diagnosis and prognosis but also as predictive biomarkers for different treatment in NSCLC patients [53,54,55,56]. The evaluation of the *EGFR* status with surgical specimens must meet a number of requirements. The specimen of tissue for nucleic acid extraction must be selected with caution and needs to correspond to a fragment rich in tumor cells while avoiding areas of strong inflammation and/or extensive sites of necrotic foci. It is often necessary to enrich the number of tumor cells by macro-dissection before the extraction of nucleic acids, notably to limit the amount of germinal DNA associated with the tumor DNA. In addition, the pre-analytical phase must be well-managed to avoid a long period of cold ischemia, which leads the nucleic acids to degrade. It is recommended that the time between surgical resection and fixation with formalin be no more than one hour [29,57]. The fixation time of the surgical specimen must be also controlled and must usually be between eight and eighteen hours depending on the size of the resected specimen [29]. It is evident that the organization of the laboratories, the surgical programs, the length of the operations, and the agendas corresponding to the surgery, in particular before a weekend or public holiday, can have a strong impact on variations to the different pre-analytical parameters. More rarely, false positive results with artifactual mutations in *EGFR* resulting from deamination due to formalin over fixation can also occur [30]. This emphasizes the importance of mastering the different pre-analytical parameters [58]. When there is a tumor heterogeneity, it is probably not necessary to look for mutations with several tissue blocks of the same resected tumor since the tumor surface for analysis is in general sufficiently large on a selected block. Nonetheless, to our knowledge, no study has verified the presence of mutations in *EGFR* on a sufficient number of tissue samples obtained from a surgical specimen to ensure the presence or absence of such a molecular heterogeneity.

The detection of mutations in *EGFR* must ideally be done in a reflex way on biopsies (or even on cytological samples) and/or on surgical specimens of all non-squamous NSCLC histological types. The majority of neoadjuvant and/or adjuvant targeted therapies are reserved for *EGFR*-mutated non-squamous carcinomas, but it is probable that these treatments will be administered in the near future for some exceptional epidermoid NSCLCs mutated on this gene [59]. Thus, this will require evaluation of mutations in *EGFR* regardless of the histological type of NSCLC [59]. Systematic analysis of the *EGFR* status on biopsies holds a number of advantages: it (i) diminishes the delay of obtaining molecular results (this is of more specific importance if neoadjuvant treatment with a targeted therapy is envisaged), (ii) avoids treatment with immunotherapy that is poorly effective and even toxic in the case of activating mutations in *EGFR*, and (iii) associates information with the molecular status of other genes of strong interest (in particular, *ALK*, *ROS1*, *NTRK*, *BRAF*, *MET*, *RET*, *NRG1*, *HER2*), which helps envisage the initiation of a potential targeted therapy, in particular in the case of recurrence or tumor progression after surgery [60,61,62,63,64,65].

## 4. Which Approach(es) for Sequencing Technology?

**Figure 2:**
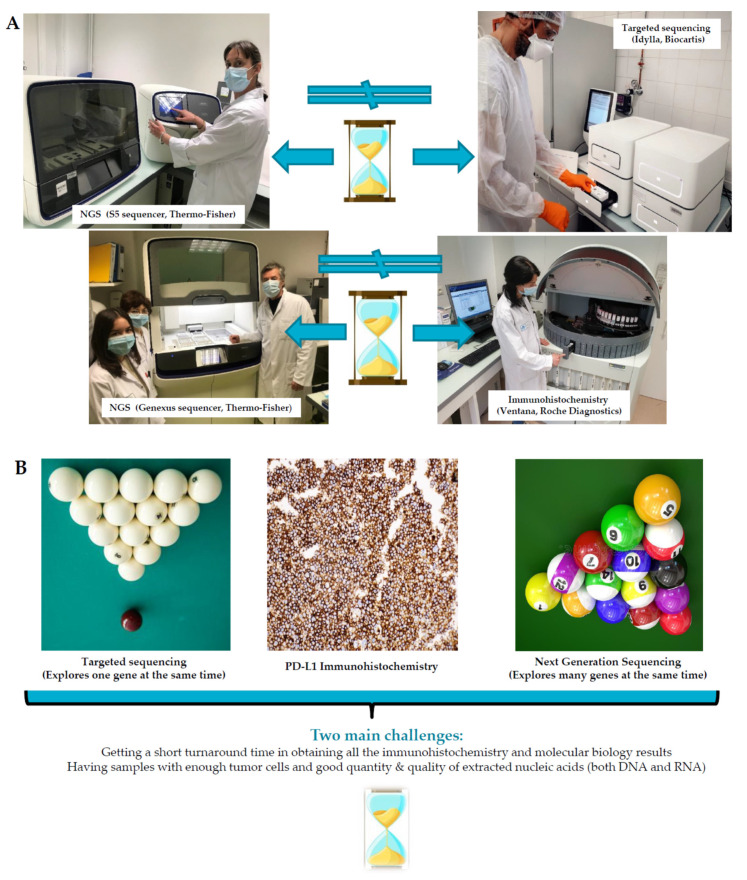
Different approaches to developing in a pathology laboratory for the assessment of predictive biomarkers in the era of neoadjuvant and/or adjuvant therapies for early stage non-small cell lung carcinoma. (**A**) Examples of different methods setup in a biopathology platform (Laboratory of Clinical and Experimental Pathology, Université Côte d’Azur, Nice, France). (**B**) Challenges associated with these different methods.

The evaluation of mutations in *EGFR* can be performed by targeted sequencing or by “next generation sequencing” (NGS) (Figure 2A,B). The use of an NGS type of approach by most of the clinical trials (for example the ADAURA trial) that target only the L858R mutation and the exon 19 deletion in *EGFR* thus seems unnecessary since this evaluation can be done by presently commercialized RT-PCR tests, notably those developed within laboratories [66,67]. Rapid tests such as those proposed by Biocartis (Mechelen, Belgium) with the Idylla approach or by Roche Diagnostics (Basel, Switzerland) using the COBAS approach can be adapted, too [66,67,68]. These latter tests can detect a large number of mutations in *EGFR*, beyond the L858R mutation and the exon 19 deletion [62,67]. In this regard, certain rare mutations in *EGFR* can be sensitive to TKIs, in particular osimertinib, and may be targeted in the future with adjuvant or neoadjuvant treatment [69]. In addition, certain rare mutations in *EGFR* may be sensitive to immuno-oncology treatment and therefore should be identified routinely [64]. Moreover, *EGFR* mutant tumors have differing responses to ICIs and underlying molecular profiles [70]. Nonetheless, interest in performing an NGS test can be strongly debated. In fact, several co-mutations associated with mutations in *EGFR* have been shown to bring about a lower efficacy to *EGFR* TKIs, to give tumor resistance to these molecules, and to result in a poorer prognosis [34,71,72,73,74,75]. This notably concerns mutations in *TP53*, *RB*, *CTNNB1*, *RBM10*, *FAT1*, *ABCB1*, *PI3KA*, and *ARID1A* [72,76,77,78,79,80]. Thus, in the future, the therapeutic strategy may differ according to these mutational associations, which make the prospective collection of this information highly indispensable. Importantly, NGS opens up future avenues to other adjuvant targeted therapeutics in the case of the detection of genomic alterations in other genes (such as *ALK*, *ROS1*, *BRAFV600*, *NTRK*, *RET*, *MET*) that are sensitive to different molecules. Thus, for example, clinical trials are ongoing for patients with operated early stage NSCLC with an *ALK* rearrangement [81]. When the status of genomic alterations is known at diagnosis, it is possible to consider this information at recurrence or tumor progression, which reduces the delay to administration of a therapeutic, in particular if it is not possible to perform an additional re-biopsy on a patient at that time. These analyses by NGS can be done on preoperative biopsies and/or surgical specimens. As for *EGFR*, the status of *ALK* must be evaluated before treatment with neoadjuvant immunotherapy and can also be evaluated by NGS, ideally by RNA sequencing [82]. It is of importance to highlight that NGS with tissue biopsies requires a sufficiently good quantity and quality of nucleic acids to obtain specific and sensitive results. In practice, it can only be used for biopsies with at least more than 20% of tumor cells (and according to the size of the tissue biopsy), which in general occurs in two out of three cases in daily practice in most situations.

## 5. Issues concerning the Cost and Reimbursement

Reliance on the sequencing strategy as a reflex test has a strong impact on the associated cost of molecular biological tests and can be discussed according to the mode of reimbursement of these tests by institutions, local organizations, and/or countries [83,84,85]. Thus, a strategy of reflex testing by NGS can be influenced by the capacity to finance these tests [67,68,86]. As an example, in France, the invoice associated with these tests is sent to the physicians (oncologists in the majority of cases) who ask for the molecular biology prescription [83]. As a matter of fact, the NGS tests are partially reimbursed in France, which incites many oncologists, notably those working in private health care systems, to prescribe RT-PCR tests for the detection of genomic alterations only associated with therapeutic molecules that have received EMA authorization and which are reimbursed [83]. Thus, some medical oncologists will most certainly prescribe only evaluation of genomic alterations in *EGFR* with respect to the administration of neoadjuvant and adjuvant therapeutics targeting activating mutations present on this gene. In fact, some studies show that the cost of NGS does not necessarily exceed the overall cost of several sequencing analyses, if analyses are not limited to the evaluation of the status of the three genes (*EGFR*, *ALK*, and *ROS1*) [87,88]. Thus, in the future, an increase in the number of therapeutic targets and genes to be examined, for care of early stage NSCLC patients, will rapidly result in a higher cost for sequential analyses compared to that of NGS, in particular with small panels of at least tens of genes [87,88]. It is obvious that the systematic setup of the reflex tests will have an impact on the workload of clinical and molecular pathology laboratories. This additional work must be considered within the network of the management of laboratories and hospitals [61]. Finally, it is well-known that the frequency of the different *EGFR* mutations in NSCLC varies according to countries and continents [89]. In this context, it is also important to consider the cost of the different testing with regard to the cost effectiveness of adjuvant targeted therapy, which has been described in some series comparatively to that of adjuvant chemotherapy [90].

## 6. Neoadjuvant Immunotherapy or Neoadjuvant Immuno/Chemotherapy versus Adjuvant Targeted Therapy against *EGFR*: How to Make the Right Choice?

Immuno-oncology clinical trials with neoadjuvant therapy (for example, the AEGEAN study, which evaluates the effect of the association of durvalumab and chemotherapy versus chemotherapy alone for resectable stage (IIA-IIIB) NSCLC, or the NEOCAST study, which evaluates the effect of durvalumab alone) or adjuvant therapy (for example, the BR.31, which evaluates the effect of durvalumab, or MERMAID-1, which evaluates the association of durvalumab and chemotherapy versus chemotherapy alone) are currently ongoing for early stage NSCLC in the absence of genomic alterations in *EGFR* and *ALK* [13,65,91]. These clinical trials need an examination of PD-L1 tumor cell expression by immunohistochemistry. These predictive biomarkers are evaluated with preoperative tissue biopsies. As an example, the AEGEAN study classifies patients according to stage II or III and to the expression of PD-L1 on tumor cells at a level below or above 1%. The biomarkers can exceptionally be evaluated on cytological material in the absence of associated tissue biopsies. The number of tumor cells in cytological samples generally allows evaluation of the expression of PD-L1; however, this assessment has to be done in more than one hundred tumor cells, which is not possible in all cases [92,93,94].

The results of biomarkers must be obtained within a delay that permits neoadjuvant treatments to be initiated, irrespective of the biological material. Thus, it is mandatory to avoid long delays in transmission of the results of PD-L1 immunohistochemistry and of the molecular biology, in particular for NGS analyses [95] (Figure 2A, B). Aside from genetic alteration in *EGFR* and *ALK*, NGS can identify other genomic alterations (in *BRAF*, *RET*, *MET*, *ROS1*, *NTRK*, *HER2*,), some of which can be harmful when neoadjuvant immunotherapy is used [96]. Given the possible intra-tumoral heterogeneity concerning the expression of PD-L1 and mutations in *EGFR* as well as some false negative results obtained with preoperative biological material, it is certainly interesting to repeat examinations for these biomarkers with surgical specimens in the case of a negative result in preoperative biopsies [97,98].

Finally, it is pivotal for these new therapeutic strategies to use robust tests for predictive biomarker (PD-L1 and *EGFR*) assessment. Thus, it has been previously demonstrated that patients with *EGFR*-mutant NSCLC showed a poor or non-response response to immunotherapy [99,100]. The mechanisms mediating this resistance of *EGFR*-mutated NSCLC patients to immunotherapy are not totally elucidated today. However, previous works revealed that *EGFR*-mutated NSCLCs have lower PD-L1 expression and a low tumor mutational burden, leading to weak immunogenicity and, thus, a weak response to ICIs [99,101]. In this context, it is crucial to set up molecular biology testing for *EGFR* evaluation, having both high specificity and sensitivity.

## 7. Perspectives and Issues

The FDA and EMA recently approved adjuvant treatment with osimertinib for early stage non-squamous NSCLC [24,25]. Thus, it is necessary to look for activating mutations in *EGFR* (L858R and deletion in exon 19) for treatment of IB-IIIA stage non-squamous lung cancer [102]. However, these investigations can certainly be applied to all operable stages including the IA stage and to all histological types of NSCLC including squamous carcinoma with the L858R mutation or the exon 19 deletion. Thus, it maybe rapidly mandatory in the near future to look for these genomic alterations in all stages and histological types of NSCLC. Rare mutations in *EGFR* can be treated with targeted therapy in adjuvant [103,104]. In contrast to certain NSCLCs, rare mutations in *EGFR* may be not sensitive to TKIs targeting these mutations, and patients may benefit better from immunotherapy associated with or without chemotherapy [70]. Moreover, the development of several third generation TKIs targeting the *EGFR* mutations opens doors to many clinical trials and new possibilities in the near future for neoadjuvant and/or adjuvant targeted therapies in early stage NSCLC [105]. Follow-up of post-operative patients with an LB can be envisaged to monitor a combination of the quantification of the circulating tumor free DNA and examination for mutations in *EGFR* as well as other associated genomic alterations [106,107]. If NGS is performed to detect mutations in *EGFR,* it is important to provide information concerning the genomic alterations present on the different genes associated with mutations in *EGFR* [34,75]. The mid- and long-term efficacy of osimertinib as a function of the different genetic signatures may in fact be different. Additionally, the ethnic origin of the patients must be taken into consideration, since it is well known that Asians present with a large percentage of early stage mutated NSCLC [108]. A recent study on Chinese patients reported a higher number of *EGFR* mutations in early stages compared to advanced stages [109]. The latter result was not observed in another study performed in patients living in the USA [75].

The sudden onset of the recent COVID-19 pandemic and the different successive waves associated with the worldwide severe acute respiratory syndrome-associated coronavirus 2 (SARS-CoV-2) infection led to a strong impact on the surgery of patients having early stage NSCLC, with a global decrease of thoracic surgical activity, an increase of delay for lung cancer resection, and certainly an increase of lung cancer mortality [110,111,112]. In the same period, a strong decrease of molecular biological activity in NSCLC patients for genomic alteration detection, including the assessment of *EGFR* mutation, was observed in most of the countries and institutions [111,113]. In this context, the number of clinical trials, notably those associated with neoadjuvant and/or adjuvant targeted therapy in early stage NSCLC, dropped, and this situation may slow down the development of these patients receiving care with different *EGFR* TKIs in the near future.

## 8. Conclusions

Pre-operative and/or post-operative treatments of NSCLC by targeted therapies or by immunotherapy (in association or not with chemotherapy) have recently revolutionized the care of early stage lung cancers and thus may prevent recurrence and progression of these tumors after surgery. Apart from the targeted therapy which has been described above, early stage NSCLC wild type for *EGFR* and *ALK* can benefit from neoadjuvant and/or adjuvant immunotherapy or immunochemotherapy in the context of ongoing large phase 3 clinical trials [3,6,12,13,17]. Therefore, these treatments provide great hope for a cure for all these cancers. However, many challenges are associated with this therapeutic revolution, not only for oncologists but also for pathologists. Thus, with the aim of providing optimal care for patients, laboratories now reflect on many pivotal questions (Table 1). Good practices and analyses of biological specimens (tissue biopsies, cytological samples, blood samples obtained before and/or after surgery, surgical specimens) strongly determine the choice of an appropriate therapy based on the evaluation of several predictive biomarkers.

It is crucial that the laboratory tests be robust (in terms of specificity and sensitivity) and the management of the biological samples be optimal, knowing that the preoperative tissue samples are becoming smaller and smaller in size, which requires great collaboration between oncologists, pathologists, and molecular biologists [29]. Moreover, for a long time, oncologists have shown interest in LB, given the number of advantages in terms of practice and facility of execution. However, while the use of these tests is more and more frequent in patients with advanced stage NSCLC, their use in patients with early stage NSCLC is still strongly debatable and open to several challenges (Table 2) [40,42].

The important nature of these novel strategies in thoracic oncology requires laboratories to ensure that the different tests are of the highest quality. Therefore, different external quality controls and accreditations according to international norms must be pursued [114]. In addition, the management of the biological tests must respect the current 2022 new legislative regulations for use (which will be applicable from May 2022), while taking into consideration the guidelines concerning the EU In Vitro Diagnostic Regulation (IVDR) [115,116,117]. By consequence, all manufacturers of in vitro diagnostic tests will be required to obtain certification to distribute the products to their clients. This will certainly offer greater standardization of molecular biology testing. However, this puts strong constraints on the working of many pathology laboratories, given the associated budgetary consequences for the setup and/or maintenance of IVDR for doing targeted sequencing and/or NGS [115,116,117].

Other different approaches that predict the response or resistance to targeted therapies and immunotherapies will certainly emerge rapidly in the future and must subsequently be integrated into the care of patients with early stage NSCLC [118,119,120,121,122]. Thus, it is certain that aside from *EGFR* and *ALK*, many other genomic alterations on other genes of interest will systematically be evaluated before immunotherapy or immunotherapy and chemotherapy to justify the therapeutic decision, while considering the benefit/risk of treatment [52]. The complexity of the biological tests will become associated with patient phenotype, genetic and epigenetic analyses, and an extension of therapeutic strategies. It is without any doubt that approaches based on algorithms that integrate developments in artificial intelligence will rapidly be envisaged in the domain of care of early stage NSCLC patients [123].

## Figures and Tables

**Table 1:** Open current and future questions concerning the evaluation of *EGFR* status of early stage NSCLC patients in the context of neoadjuvant and/or adjuvant therapies using *EGFR* TKIs.

Should we only look for the *EGFR* status on pre-operative biopsies (bronchial and transthoracic biopsies)?
Should we only look for the *EGFR* status on surgically resected specimens?
Should we look for the *EGFR* status systematically on both biopsies and surgically resected specimens?
Is it acceptable to look for the *EGFR* status on cytological samples only?
What is the added value of integrating a liquid biopsy before and/or after surgery for early stage NSCLC patients?
Does the assessment of *EGFR* have to be done only by targeted (RT-PCR) or next generation sequencing technologies?
What about the systematic evaluation of the other predictive biomarkers on biopsies (PD-L1, *ALK*, and other genes)?
How can we master the turnaround time in obtaining results, notably in the context of neoadjuvant immunotherapy versus adjuvant *EGFR* TKIs administration?
How should we consider the landscape of concomitant mutation (notably in *TP53*, *RB*, *RBM10*, *CTNNB1*, *FAT1*, *ABCB1*, *ARID1A*) in *EGFR* 19 del19/L858R mutated tumors and their association with response to *EGFR*-TKIs in early stage NSCLC patients?
How should we consider *EGFR* mutations out of *EGFR* 19 del19/L858R?
How should we take care of patients with early stage squamous cell lung carcinoma with an *EGFR* mutation?
How should we reimburse the molecular biology reflex tests and receive coverage for the full cost?
How should we integrate the induced workload and new infrastructural challenges in the pathology laboratories?
How should we anticipate the issues associated with the development of the In Vitro Diagnostic Regulation in Europe?

**Table 2:** Main challenges facing liquid biopsies for early stage NSCLC patients.

Should we assess the ct-DNA level before and/or after surgery?
What is the best timing for blood sampling after complete surgery?
How should we define a cutoff for the ct-DNA level after complete surgery?
How should we set up a liquid biopsy in new daily practice?
How should we determine false and true negative results for *EGFR* mutation in a liquid biopsy?
How should we determine false positive results for *EGFR* mutation in a liquid biopsy?
What are the best practices for mastering the different pre-analytical phases?
How should we distinguish cf-DNA and ct-DNA in routine clinical practice?
How should we set up blood methods for standardization and validation of molecular biology testing?
How should we obtain a mandatory accreditation (such as the ISO 15,189 accreditation in Europe)?
How should we reimburse the liquid biopsy in the health care system?

## Data Availability

Not applicable.
